# Identification of Amplified Fragment Length Polymorphism (AFLP) Markers Tightly Associated with Drought Stress Gene in Male Sterile and Fertile *Salvia miltiorrhiza* Bunge

**DOI:** 10.3390/ijms14036518

**Published:** 2013-03-22

**Authors:** Yuejin Zhang, Lijun Guo, Zhiming Shu, Yiyue Sun, Yuanyuan Chen, Zongsuo Liang, Hongbo Guo

**Affiliations:** Shaanxi Research Center of TCM Fingerprinting and NP Library, College of Life Sciences, Northwest A&F University, Yangling 712100, Shaanxi, China; E-Mails: zhangxyj@nwsuaf.edu.cn (Y.Z.); guolijunbenben@yahoo.com.cn (L.G.); shuzhiming2298@yahoo.com.cn (Z.S.); sunyiyue2005@163.com (Y.S.); yuanyuan2011051433@163.com (Y.C.); liangzs@ms.iswc.ac.cn (Z.L.)

**Keywords:** AFLP, bulked segregant analysis, drought stress, male sterility, near-isogenic lines, *Salvia miltiorrhiza*, water deficit, water stress

## Abstract

Consistent grain yield in drought environment has attracted wide attention due to global climate change. However, the important drought-related traits/genes in crops have been rarely reported. Many near-isogenic lines (NILs) of male sterile and fertile *Salvia miltiorrhiza* have been obtained in our previous work through testcross and backcross in continuous field experiments conducted in 2006–2009. Both segregating sterile and fertile populations were subjected to bulked segregant analysis (BSA) and amplified fragment length polymorphism (AFLP) with 384 and 170 primer combinations, respectively. One out of 14 AFLP markers (E9/M3_246_) was identified in treated fertile population as tightly linked to the drought stress gene with a recombination frequency of 6.98% and at a distance of 7.02 cM. One of 15 other markers (E2/M5_357_) was identified in a treated sterile population that is closely associated with the drought stress gene. It had a recombination frequency of 4.65% and at a distance of 4.66 cM. Interestingly, the E9/M3_246_ fragment was found to be identical to another AFLP fragment E11/M4_208_ that was tightly linked to the male sterile gene of *S. miltiorrhiza* with 95% identity and *e*-value 4 × 10^−93^. Blastn analysis suggested that the drought stress gene sequence showed higher identity with nucleotides in *Arabidopsis* chromosome 1–5.

## 1. Introduction

Crops are often subjected to periods of soil and atmospheric water deficits during their growth cycle in many regions of the globe. The faster-than-predicted climate change and different available scenarios are increasing the aridity of the semi-arid regions, which will lead to an over-exploitation of water resources for agricultural purposes, increased constraints to crop growth and survival and consequently to realizing crop yield [1]. It was estimated by the National Drought Mitigation Center that more than 1.3 billion dollars in crop losses occurred due to the 2007 drought in the southeastern United States [2]. Understanding the tendency associated with drought at the regional scale could provide useful insights for farmers and cultivar breeders to reduce their losses. Shaanxi Province is located in northwestern China within a semi-arid and arid region. Since 1961, minimum rain fall has increased considerably over Shaanxi Province and the intensity is becoming more severe [3].

The root and shoot of *Salvia miltiorrhiza* Bunge are used as a traditional Chinese herb drug for removing blood stasis, alleviating pain, promoting the circulation of blood, promoting menstruation, tranquilizing the brain, and treating cardiovascular and cerebrovascular disease [4]. Although our previous research showed that severe drought stress could increase the contents of most of the active constituents in *S. miltiorrhiza*, both root and shoot dry weight were significantly decreased [5]. This was also the case in wheat and rice [6,7]. Moreover, until now, there has been no hybrid cultivar with drought tolerance to *S. miltiorrhiza*, which is disadvantageous to quality control of medicinal materials even for those individuals growing in one field under the same intensity of drought stress.

Since a natural male sterile mutant of *S. miltiorrhiza* (Sh-B) was first found in 2002, research has been conducted to determine its pollen development [8], biological characteristics [9] and field hybrid experiment [10], through which many near-isogenic lines (NILs) and hybrid combinations were obtained. Breeding hybrid cultivar with drought tolerance from NILs would be important for farmers to harvest normal yield with qualified quality of medicinal material under drought-stress environment. Amplified fragment length polymorphism (AFLP) technique is one of the most efficient molecular marker systems for screening genes of interest [10–12]. In our previous work we have successfully screened one AFLP marker that tightly linked with the dominant male sterile gene in *S. miltiorrhiza*[10] and the marker has been applied to marker-assisted selection (MAS) of the male sterile population.

The aims of this investigation were: (1) to screen AFLP markers that tightly associate with drought stress gene in both male sterile and fertile NILs of *S. miltiorrhiza*; (2) to examine the fertility change for fertile plants under severe drought stress conditions.

## 2. Results and Discussion

### 2.1. Identification of AFLP Marker Linked to Drought Stress Gene in Fertile S. miltiorrhiza

**Figure 1:**
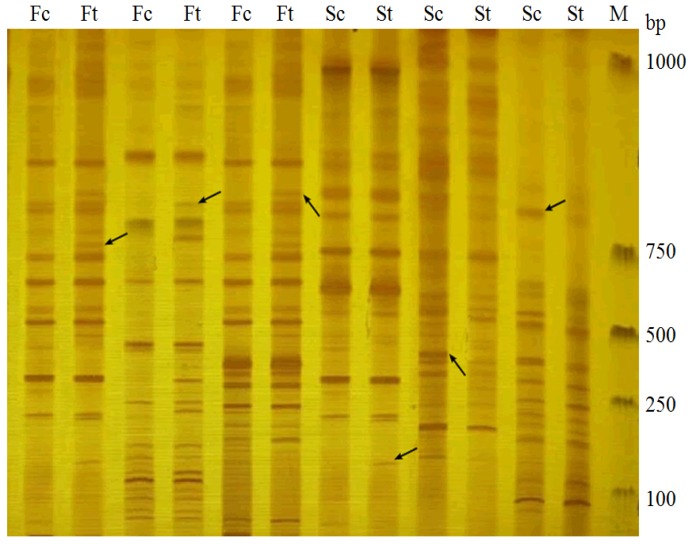
Selective amplification in fertile control (Fc), drought stress treated fertile (Ft), sterile control (Sc) and drought stress treated sterile (St) near-isogenic lines of *Salvia miltiorrhiza* populations by amplified fragment length polymorphism (AFLP). Arrows represent differently expressed bands between them.

In this analysis, the control fertile and treated fertile bulks were used to identify putative markers linked to drought stress gene. The assays involved two common enzymes (EcoRI and MseI). A total of 170 pairs of primer combinations were used with E+3/M+3. All primer combinations amplified 5204 fragments with an average of 30.60. Fourteen primer combinations revealed reproducible polymorphism (8.20%) between the fertile and treated DNA bulks after three PCR amplification replications (Figure 1).

**Figure 2:**
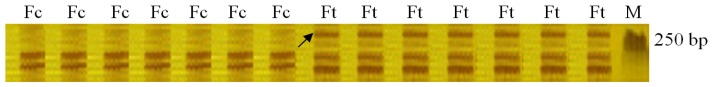
AFLP amplification profiles in fertile control (Fc) and drought stress treated fertile (Ft) near-isogenic line of *Salvia miltiorrhiza* population generated by primer combination E9/M3_246_. The arrow represents the band that tightly linked with drought stress gene in Ft population of *S. miltiorrhiza*.

Verified examination of eight fertile and eight treated individuals in the bulks indicated that one of 14 AFLP markers, E9/M3_246_ (E9: 5′-GACTGCGTACCAATTCACC-3′, M3: 5′-GATGAGTCCTGAGTAACAG-3′), was associated with drought stress gene (Figure 2). This AFLP marker was confirmed in the 45 fertile and 44 stress treated fertile plants and then was cloned and sequenced. Linkage analysis confirmed that this AFLP marker was tightly linked to the drought stress gene with a recombination frequency of 6.98% and at a distance of 7.02 cM.

### 2.2. Identification of AFLP Marker Linked to Drought Stress Gene in Sterile S. miltiorrhiza

In this part, both control sterile and treated sterile bulks were used to identify putative markers linked to drought stress gene, in which both EcoRI and MseI enzymes were involved. A total of 384 pairs of primer combinations were employed and 8254 fragments were amplified with an average of 27.3. Fifteen primer combinations exhibited reproducible polymorphism (5.0%) between male sterile and treated bulks after three-replication PCR amplifications (Figure 1).

**Figure 3:**
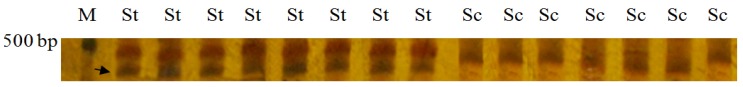
AFLP amplification profiles in sterile control (Sc) and drought stress treated sterile (St) near-isogenic line of *Salvia miltiorrhiza* population generated by primer combination E2/M5_357_. The arrow represents the band that is tightly linked with drought stress gene in St population of *S. miltiorrhiza*.

Re-examination of eight sterile and eight treated plants in the bulks showed that one of 15 markers, E2/M5_357_ (E2: 5′-AACGGGCTTGGAAACGATGG-3′, M5: 5′-CTGTGCCAATGCGAATGCTC-3′), was linked with drought stress gene (Figure 3). This AFLP marker was verified in 46 sterile and 45 treated sterile individuals and then was cloned and sequenced. Linkage analysis confirmed that this AFLP marker was tightly associated with the drought stress gene with a recombination frequency of 4.65% and at a distance of 4.66 cM.

### 2.3. Sequence Features of Drought Stress Gene Amplified by E9/M3 and E2/M5 Primer Combinations

Two fragments (246 and 357 bp) amplified by E9/M3 and E2/M5 primer combinations, respectively, were submitted to the NCBI website (http://ncbi.nlm.nih.gov/) for nucleotide-nucleotide BLAST (Blastn) analysis. The sequences were identical to nucleotides in *Arabidopsis* genome ranging from 95% to 100% and 91% to 100%, respectively, and in *Oryza sativa* japonica genome with the percentage of 92%–93% and 92%–100%, respectively (Table 1).

Interestingly, the fragment (246 bp) tightly linked with drought stress gene in the treated fertile population was identical to another AFLP fragment (208 bp) that was proved to be tightly associated with the male sterile gene in the sterile population [10] with identity 95% and *e*-value 4 × 10^−93^ (Table 1). Although both fragments shared the same primer combination, the result suggested that drought stress might cause the male sterility in fertile *S. miltiorrhiza*.

### 2.4. Discussion

To date, there is no known trait/gene in medicinal plant species that has been mapped using molecular markers. Bulked segregant analysis (BSA) combined with amplified AFLP methodology was firstly employed to identify markers linked to the male sterile [10] and drought stress gene of *S. miltiorrhiza*. BSA has been proven to be an efficient and rapid method to detect markers in specific genomic regions that are linked to a target gene or trait [10,11,13,14]. Similarly, many AFLP markers were shown to tightly link to different genes/traits [10,11,14].

Our results showed that the fragment (246 bp) linked to the drought stress gene in the stress-treated fertile population was identical to another fragment (208 bp) that tightly associated with the male sterile gene in the male sterile population with identity 95% and *e*-value 4 × 10^−93^ by using the same primer combination [10]. This indicates that fertile NIL population of *S. miltiorrhiza* loses its fertility after one-month severe drought stress when meiosis and the young microspore stage are included. During meiosis and at the young microspore stage the tapetum is metabolically extremely active [15], but would cause a premature cell death (PCD) response when meeting drought stress [16]. In male gametophyte of male sterile *S. miltiorrhiza*, tapetal hypertrophy was observed as well as the delayed natural programmed cell death [8].

The fragment (208 bp) linked with male sterile gene in *S. miltiorrhiza* exhibited 100% identity to *S*-adenosylmethionine-dependent methyltransferase domain-containing protein in *Arabidopsis thaliana* chromosome 1 with *e*-value 5.2 [10]. The identical fragment (246 bp) obtained in this paper also showed 100% identity with this domain at 3′ end (Table 1). *S*-adenosylmethionine (AdoMet) is a common co-substrate involved in methyl metabolic pathways including transmethylation, transsulfuration and aminopropylation. Methylation of genomic DNA is an epigenetic regulatory mechanism involved in controlling transcriptional regulation and chromatin structures that is required for meiosis and post-meiotic maturation [17]. DNA methylation has been proved to be involved in the development of plant gametogenesis [18,19].

The 246 bp fragment also showed 100% identity to 386 bp at 3′ side of mitochondrial transcription termination factor (mTERF) protein family near the 3′ end with *e*-value 0.52 (Table 1). mTERF protein family locates in mitochondria. mTERF2, one of the members, has been found to bind with mitochondrial DNA that showed negative effect on mtDNA replication and down-regulated all the oxidative phosphorylation components in the mitochondria, which is essential for energy metabolism [20]. Several mitochondrial regulators encoded by the nuclear genome have been identified [21]. During meiosis and the young microspore stage, the number of mitochondria per tapetum cell increased 20–40-fold in order to satisfy the high energy demand [22]. However, the important functions of tapetum in this stage would be interfered under drought conditions and undergo a senescence response restricting sugar accumulation.

Stress-induced pollen sterility is not only restricted to monocots, but also occurs in dicot plants. Irreversible abortion of pollen development is found in both rice and wheat when induced by drought stress at the young microspore stage [23,24]. Both cold and drought stresses were shown to trigger a PCD response in the tapetum due to rising levels of reactive oxygen species in conjunction with a down regulation of antioxidant systems [25,26] and caused abscisic acid (ABA) accumulation [27]. ABA is proved to play important roles in male sterility of tomato (*Solanum lycopersicum*) and in interactions with sugar signaling [28]. The fact that ABA down-regulates cell wall invertase [7] activity in the vascular parenchyma cells, which is proposed as the cause of sugars shortages and may be the main reason for the abortion of pollen development [29]. The ABA biosynthesis was controlled by the gene encoding zeaxanthin epoxidase (ZEP) which participates xanthophylls cycles in higher plants [30]. The 208 bp fragment linked with male sterile gene in *S. miltiorrhiza* showed also showed 95% identity to ZEP with *e*-value 5.2 at 5′ end [10].

The fragment (357 bp) linked to drought gene in treated sterile *S. miltiorrhiza* exhibited 100% identity to ATP-binding cassette (ABC) transporter B family member 16 (Table 1). The ABC superfamily comprises both membrane-bound transporters and soluble proteins involved in a broad range of processes, many of which are of considerable agricultural, biotechnological and medical potential [31]. The large and diverse subfamily B is defined by domain organization and sequence homology to prototypes from humans and yeasts. The full length P-glycoprotein multidrug resistance transporters have evidently undergone appreciable functional diversification in plants, assuming roles in the transport of auxins, secondary metabolites and xenobiotics [32].

The 357 bp fragment also showed 100% identity to flavin-binding monoxygenase family with *e*-value 2.7 (Table 1). The YUC family of flavin monooxygenase genes plays important roles in auxin biosynthesis and disruption of the YUC genes in *Arabidopsis* leads to defects in floral development, vascular tissue formation and other developmental processes [33]. Auxin has been suggested to play a critical role in vascular patterning and its overproduction in a whole plant would increase the amount of vascular tissue [34], which may be a reason for tapetal hypertrophy observed in sterile *S. miltiorrhiza* male gametophyte.

Our previous results showed that drought stress significantly decreased both shoot and root dry weight in fertile *S. miltiorrhiza*, but increased the root to shoot ratio at later growth cycle [5]. Increasing root to shoot ratio and closing stomata are the plant strategy to maximize water uptake and minimize water loss in response to water stress, in which *OsCOW1*- mediated IAA biosynthesis is involved in rice root [35]. The effect of drought on leaf photosynthesis may be direct, as the decreased CO_2_ availability caused by diffusion limitations through the stomata and the mesophyll or the alterations of photosynthetic metabolism or they can arise as secondary effects, namely oxidative stress [1].

It is loss in grain number, rather than a reduction in grain size, that largely accounts for crop yield reduction when abiotic stress occurs [36]. This situation may be overcome by using NIL or populations only differing in target trait, gene or QTL. Although medium and severe drought stress decreased root dry weight 29% and 39% in fertile *S. mitiorrhiza*, respectively [5], the root yield of F1 hybrid is found not to decrease but to slightly increase when using NILs as parent. Both root and shoot of one-year-old sterile individuals are smaller than the same old fertile ones, but both traits in the two-year-old sterile and fertile plants are similar. Despite the fact that many crops harvest grain but not roots, hybrid heterosis may partially reduce the negative drought effect on grain yield.

## 3. Experimental Section

### 3.1. Plant Material

Both NILs of male sterile and fertile *S. miltiorrhiza* were obtained from our previous field hybrid experiment from 2006 to 2009 [10]. All materials were grown on the experimental farm of Northwest A&F University for collection and resources conservation.

### 3.2. Drought stress Treatments

A pot experiment was carried out from April to May 2011 with 24 cm (diameter) × 22 cm (height). Both soil and clean sand (2:1, *v*/*v*) were filled in pot after passing through a 0.5 cm mesh sieve. Four-month-old seedlings collected from an experiment farm were transplanted into each pot with three individuals. Each treatment was replicated 20 times.

The drought stress experiment included two treatments, control and severe drought stress (40% field water capacity). All pots were placed under a rain shelter. Each pot was weighed at 6 p.m. every day and then the necessary water loss was calculated. Young leaf samples were collected after one-month treatment for DNA extraction.

### 3.3. AFLP Fragment Sequencing and Linkage Analysis

Fresh young leaves of *S. miltiorrhiza* from randomly selected plants in each treatment were collected and DNA was extracted by using our improved cetyltrimethylammonium bromide (CTAB) method [10]. For bulked segregant analysis (BSA, [13]), equivalent amounts of DNA from eight randomly selected sterile individuals (control) and eight treated sterile ones were pooled to construct sterile and treated bulks, respectively. The same amount of DNA from eight fertile plants (control) and eight treated fertile ones were used to construct fertile and treated fertile bulks, respectively.

The AFLP procedure, fragment cloning and sequencing were followed by Shu *et al.*[10]. The homology of the sequenced AFLP markers linked to drought stress gene was determined using BLASTn by comparison with the database at NCBI [37].

Those exhibiting reproducible and polymorphic AFLP markers between the control and treated bulks were identified in the NILs mapping population. The drought stress gene and AFLP marker data were combined for linkage analysis using the software package MAPMAKER/EXP 3.0 [38,39]. The recombinant frequencies between drought stress gene and AFLP markers were calculated through two-point tests and a linkage map was constructed by three-point or multi-point tests with a minimum LOD threshold of 3.0. The recombination values were converted into centiMorgans (cM) by using the Kosambi mapping function [40].

## 4. Conclusions

In conclusion, two AFLP markers (E9/M3_246_ and E2/M5_357_) were identified in treated fertile and treated sterile NILs of *Salvia miltiorrhiz*, respectively, both tightly linked to the drought stress trait/gene. The E9/M3_246_ fragment was found to be identical to another AFLP fragment E11/M4_208_ that tightly linked to male sterile gene of *S. miltiorrhiza* with 95% identity and *e*-value 4 × 10^−93^.

## Figures and Tables

**Table 1:** Both blast hits of AFLP fragments E9/M3_246_ and E2/M5_357_ that tightly linked with drought stress gene in stress-treated fertile and stress-treated sterile *Salvia miltiorrhiza*, respectively.

GenBank Acc. No.	Blast hits	Organism	*e*-value	Query cover (%)	Identity (%)
E9/M3_246_					

NC003071.7	Hypothetical protein	*Arabidopsis thaliana*, chromosome 2	0.15	32	100
NC003074.8	Hypothetical protein	*Arabidopsis thaliana*, chromosome 3	0.52	23	100
NC003070.9	1536 bp at 5′ side: hypothetical protein; 386 bp at 3′ side: mitochondrial transcription termination factor family protein	*Arabidopsis thaliana*, chromosome 1	0.52	39	100
NC003076.8	Putative leucine-rich repeat receptor-like protein kinase	*Arabidopsis thaliana*, chromosome 5	6.3	17	95
NC008396.2	Hypothetical protein	Oryza sativa Japonica Group DNA Chromosome 3	0.47	10	93
NC008398.2	38938 bp at 5′ side: hypothetical protein; 11051 bp at 3′ side: hypothetical protein	Oryza sativa Japonica Group DNA Chromosome 5	1.7	19	92
NC008399.2	23149 bp at 5′ side: hypothetical protein; 2957 bp at 3′ side: hypothetical protein	Oryza sativa Japonica Group DNA Chromosome 6	5.8	10	92

E2/M5_357_					

NC003074.8	ABC transporter B family member 16	*Arabidopsis thaliana*, chromosome 3	0.22	26	100
NC003076.8	DNA-3-methyladenine glycosylase I	*Arabidopsis thaliana*, chromosome 5	2.7	12	91
NC003075.7	Hypothetical protein	*Arabidopsis thaliana*, chromosome 4	2.7	12	100
NC003071.7	2397 bp at 5′ side: hypothetical protein; 1525 bp at 3′ side: laccase-4	*Arabidopsis thaliana*, chromosome 2	2.7	31	95
NC003070.9	18 bp at 5′ side: flavin-binding monooxygenase family protein; 462 bp at 3′ side: putative F-box/kelch-repeat protein	*Arabidopsis thaliana*, chromosome 1	2.7	29	100
NC008404.2	17867 bp at 5′ side: hypothetical protein; 46979 bp at 3′ side: hypothetical protein	Oryza sativa Japonica Group DNA Chromosome 11	2.5	14	92
NC008397.2	Hypothetical protein	Oryza sativa Japonica Group DNA Chromosome 4	2.5	5	100
NC008395.2	Hypothetical protein	Oryza sativa Japonica Group DNA Chromosome 2	2.5	18	95

E2/M5_357_					

NC008403.2	Hypothetical protein	Oryza sativa Japonica Group DNA Chromosome 10	8.7	5	100
NC008402.2	2747 bp at 5′ side: hypothetical protein; 1409 bp at 3′ side: hypothetical protein	Oryza sativa Japonica Group DNA Chromosome 9	8.7	5	100
E9/M3_246_ and E11/M4_208_	–	–	4 × 10^−93^	84	95
E2/M5_357_ and E11/M4_208_	–	–	0.03	3	100
E9/M3_246_ and E2/M5_357_	–	–	0.036	4	100
